# Author Correction: Central American mountains inhibit eastern North Pacific seasonal tropical cyclone activity

**DOI:** 10.1038/s41467-026-68407-2

**Published:** 2026-03-16

**Authors:** Dan Fu, Ping Chang, Christina M. Patricola-DiRosario, R. Saravanan, Xue Liu, Hylke E. Beck

**Affiliations:** 1 International Laboratory for High-Resolution Earth System Prediction, Texas A&M University, College Station, TX USA; 2 Department of Oceanography, Texas A&M University, College Station, TX USA; 3 Department of Atmospheric Sciences, Texas A&M University, College Station, TX USA; 4 Department of Geological and Atmospheric Sciences, Iowa State University, Ames, IA USA; 5 Climate and Ecosystem Sciences Division, Lawrence Berkeley National Laboratory, Berkeley, CA USA; 6 Department of Civil and Environmental Engineering, Princeton University, Princeton, NJ USA

Correction to: *Nature Communications* 10.1038/s41467-021-24657-w, published online 20 July 2021

Since the publication of this work, Christina M. Patricola-DiRosario has changed their name from Christina M. Patricola. This has now been amended in the HTML and PDF versions of the article.

