# Supplementary material for: Central American mountains inhibit eastern North Pacific seasonal tropical cyclone activity
Source: Nat Commun. 2021 Jul 20;12:4422. doi: 10.1038/s41467-021-24657-w (PMC8292387; doi:10.1038/s41467-021-24657-w)
Supplement: Supplementary file 1 — Supplementary Information [file 41467_2021_24657_MOESM1_ESM.pdf]

Central American mountains inhibit eastern North Pacific seasonal tropical cyclone activity

Dan Fu[1,2,*], Ping Chang[1,2,3], Christina M. Patricola[4,5*], R. Saravanan[1,3],

Xue Liu[1,2] and Hylke E. Beck[6]

1. International Laboratory for High-Resolution Earth System Prediction, Texas A&M University, College Station, Texas, USA
2. Department of Oceanography, Texas A&M University, College Station, Texas, USA.
3. Department of Atmospheric Sciences, Texas A&M University, College Station, Texas, USA.
4. Department of Geological and Atmospheric Sciences, Iowa State University, Ames, Iowa, USA.
5. Climate and Ecosystem Sciences Division, Lawrence Berkeley National Laboratory, Berkeley, California, USA.
6. Department of Civil and Environmental Engineering, Princeton University, Princeton, New Jersey, USA
* Corresponding authors email: fudan1991@tamu.edu or cmp28@iastate.edu

Supplementary Figures

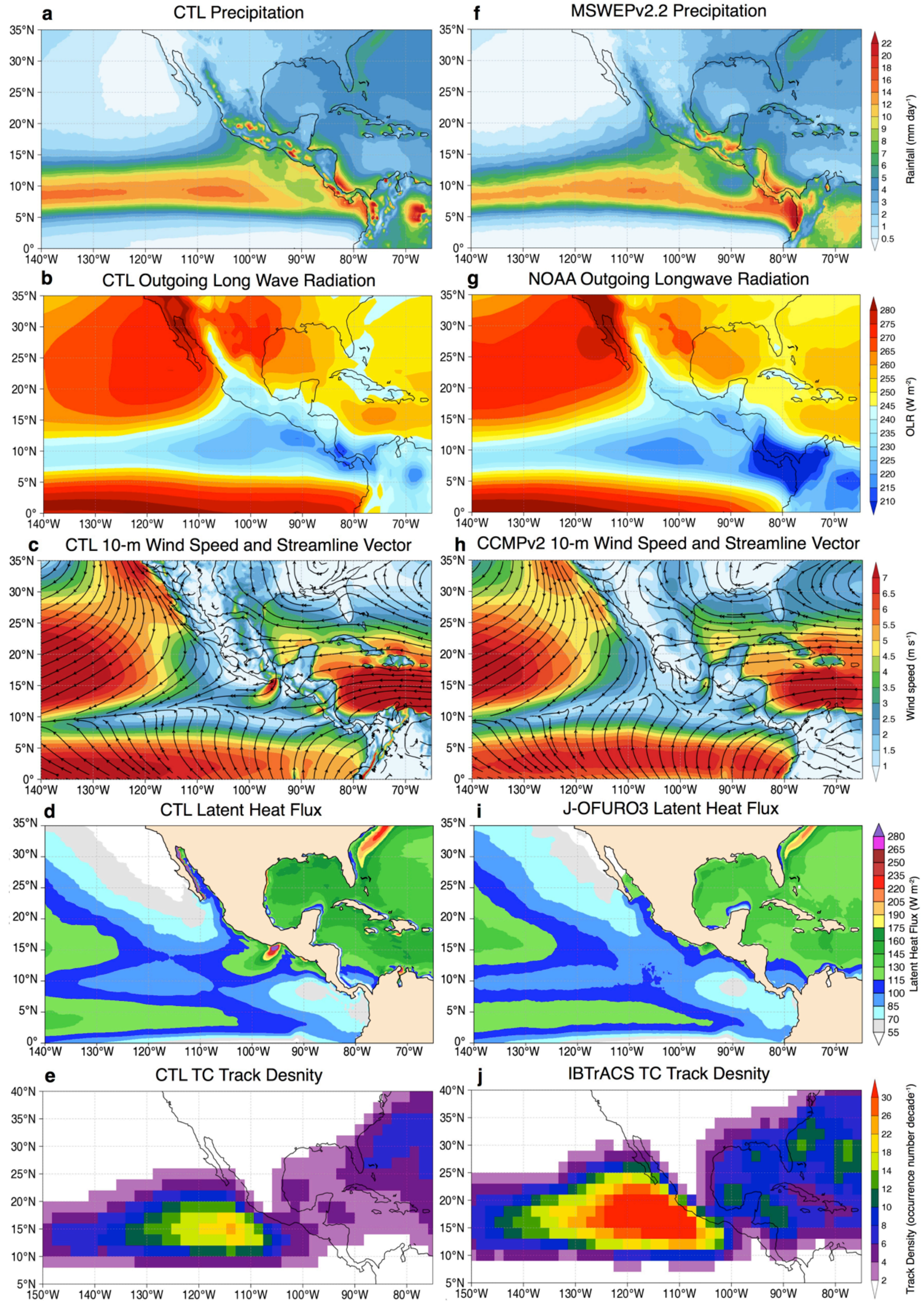

Supplementary Figure 1 | Validation of the CTL simulation. Seasonal averaged a) precipitation (mm day^{-1}), b) outgoing longwave radiation (OLR; W m^{-2}), c) 10-m wind (m s^{-1}), d) latent heat flux (W m^{-2}), and e) TC track density (occurrence number $decade^{-1}$), from the CTL simulation ensemble mean. f-j) Are similar, but from various observational datasets (refer subtitles for details).

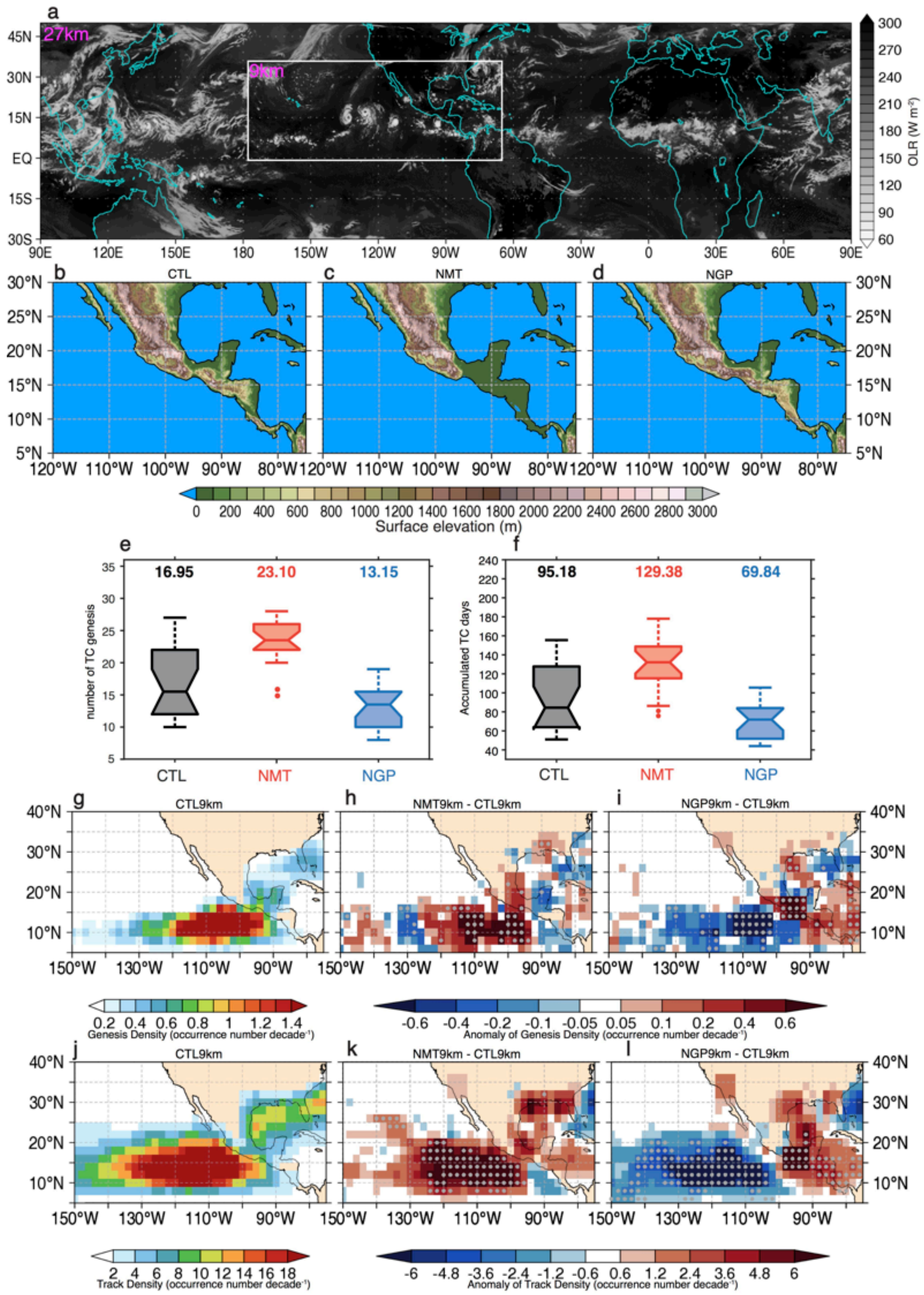

Supplementary Figure 2 | Model domain and topography for nested 9 km simulation. a) A snapshot of the simulated OLR (W m^{-2}). The outer domain has horizontal resolution of 27 km (1484×377 grid boxes), while the one-way nested inner domain (white box) is configured with 9 km horizontal resolution (1399×496 grid boxes). Three simulated TCs are notable, one landfalling over the South China and a pair of twin TCs over the ENP. b-d) The topography (m) used in 9 km inner domain for the 30-member ensemble CTL9km, NMT9km and NGP9km simulations, respectively. e-l) Similar to Figure 1 d-k), but for the 9 km model simulation results.

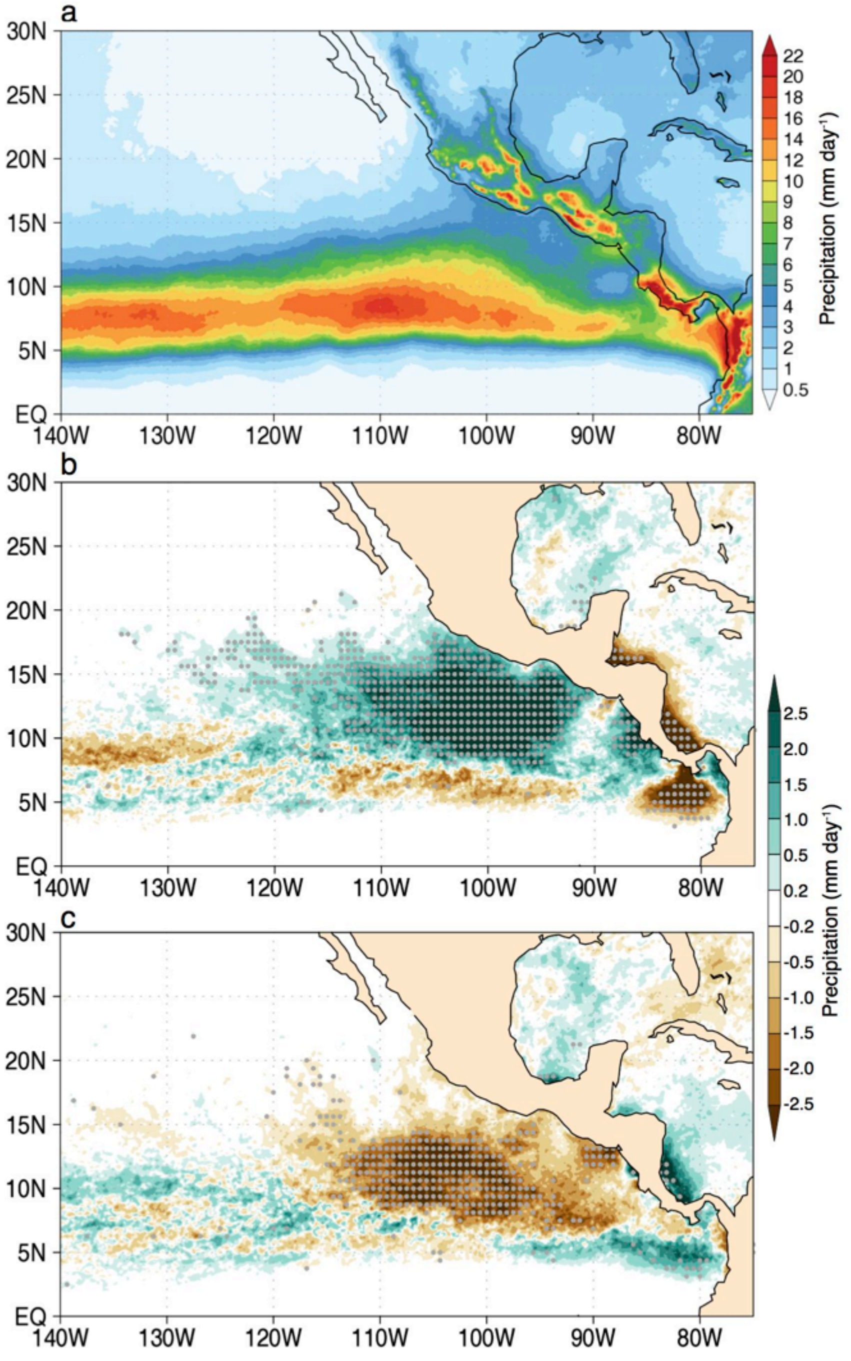

Supplementary Figure 3 | Precipitation response to topography derived from nested 9 km simulation. a) June-November 30-member ensemble averaged precipitation (mm day^{-1}) from 9km CTL simulation. The difference of precipitation (mm day^{-1}) using 30-member ensemble mean of the a) NMT and b) NGP minus CTL. Grey dots denote statistical confidence at the 95% level based on the two-sided t-test.

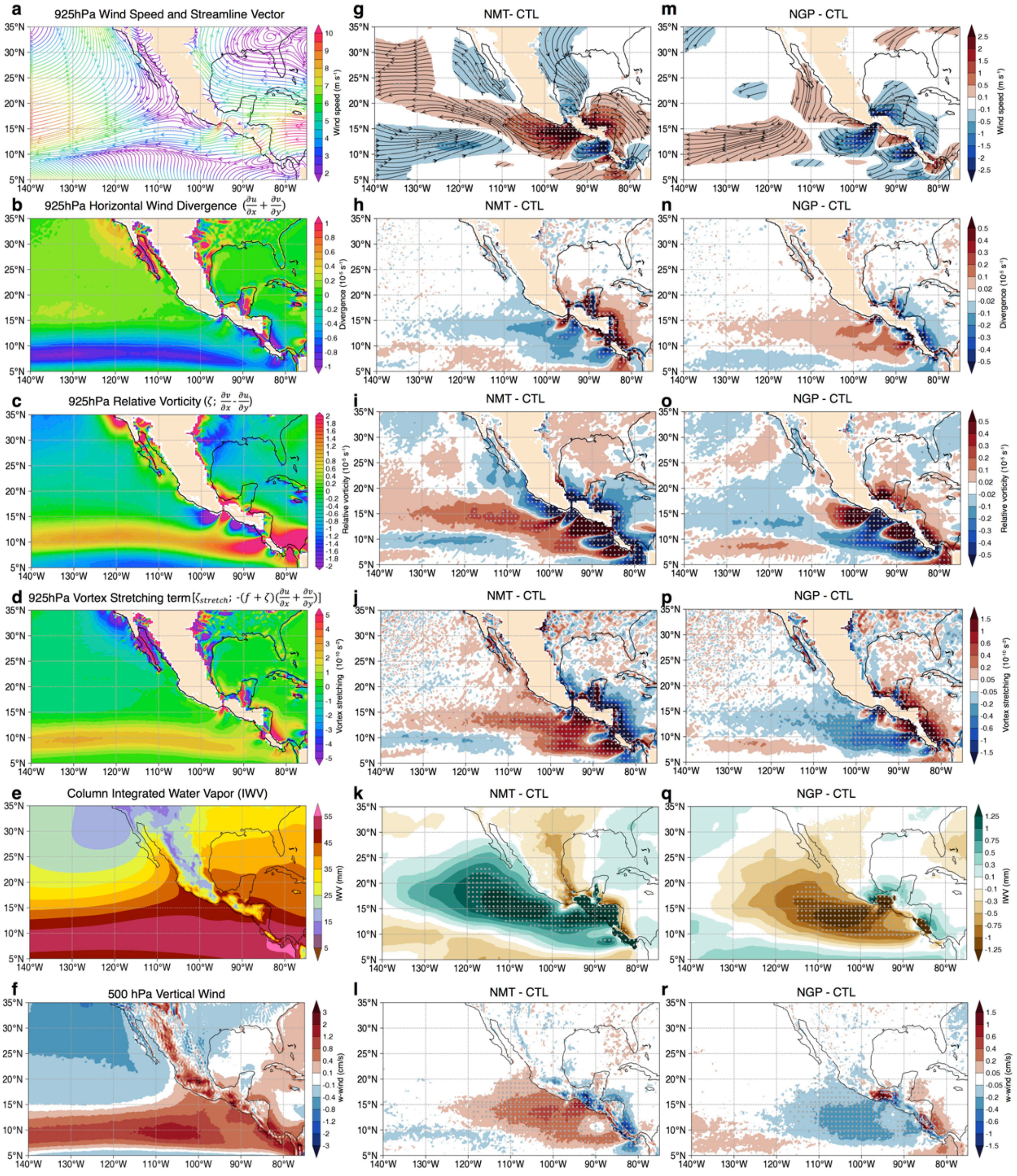

Supplementary Figure 4 | Topography-induced responses of mean circulation. Seasonal averaged a) 925hPa wind speed (m s^{-1}), b) 925hPa horizontal wind divergence (10^{-5} s^{-1}), c) 925hPa relative vorticity (10^{-5} s^{-1}), d) 925 hPa vorticity equation vortex stretching term [$-(f+\zeta)(\partial u/\partial y+\partial v/\partial y)$; 10^{-10} s^{-2}], e) column integrated water vapor (IWV; mm), and f) 500 hPa vertical wind (cm s^{-1}) from the CTL simulation ensemble mean. g-l) And m-r) are similar, but from the differences using the NMT minus CTL and NGP minus CTL, respectively. Grey dots denote statistical confidence at the 95% level based on the two-sided t-test. Light orange shadings indicate the mountain outlines at 925 hPa.

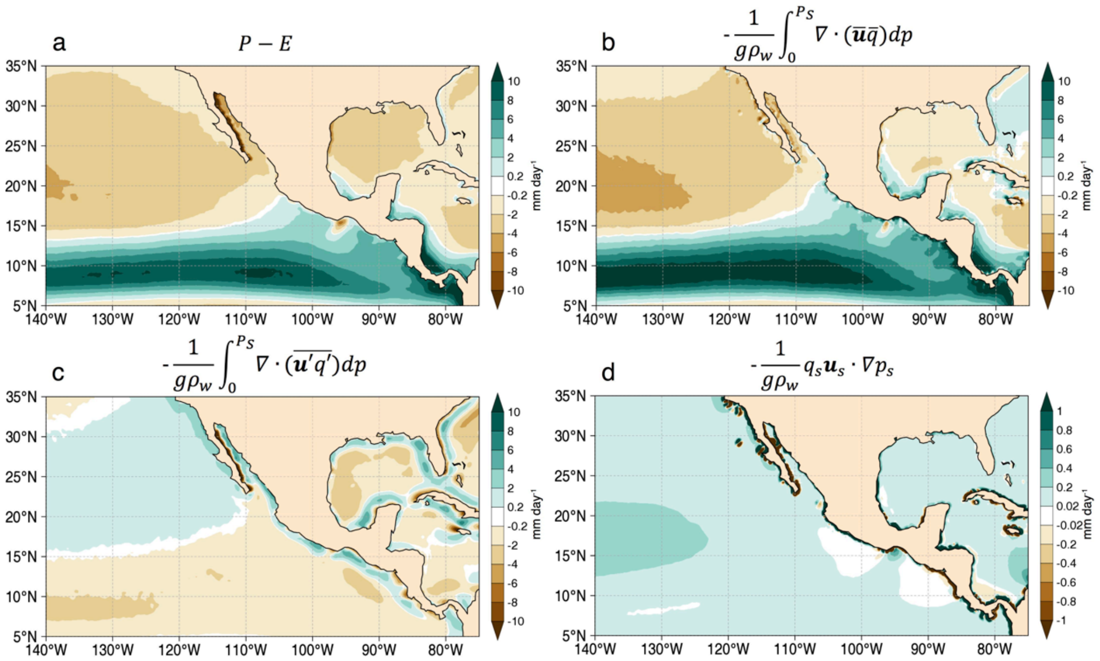

Supplementary Figure 5 | Moisture budget terms from the CTL simulation ensemble mean. a) Precipitation minus evaporation (P-E; mm day^{-1}), b) moisture flux convergence by the monthly mean flow (mm day^{-1}), c) moisture flux convergence by the transient eddy (daily mean departures from the monthly mean; mm day^{-1}) and d) surface quantities (mm day^{-1}) term. Refer the Methods section for the details of mositure budget equation and decomposition.

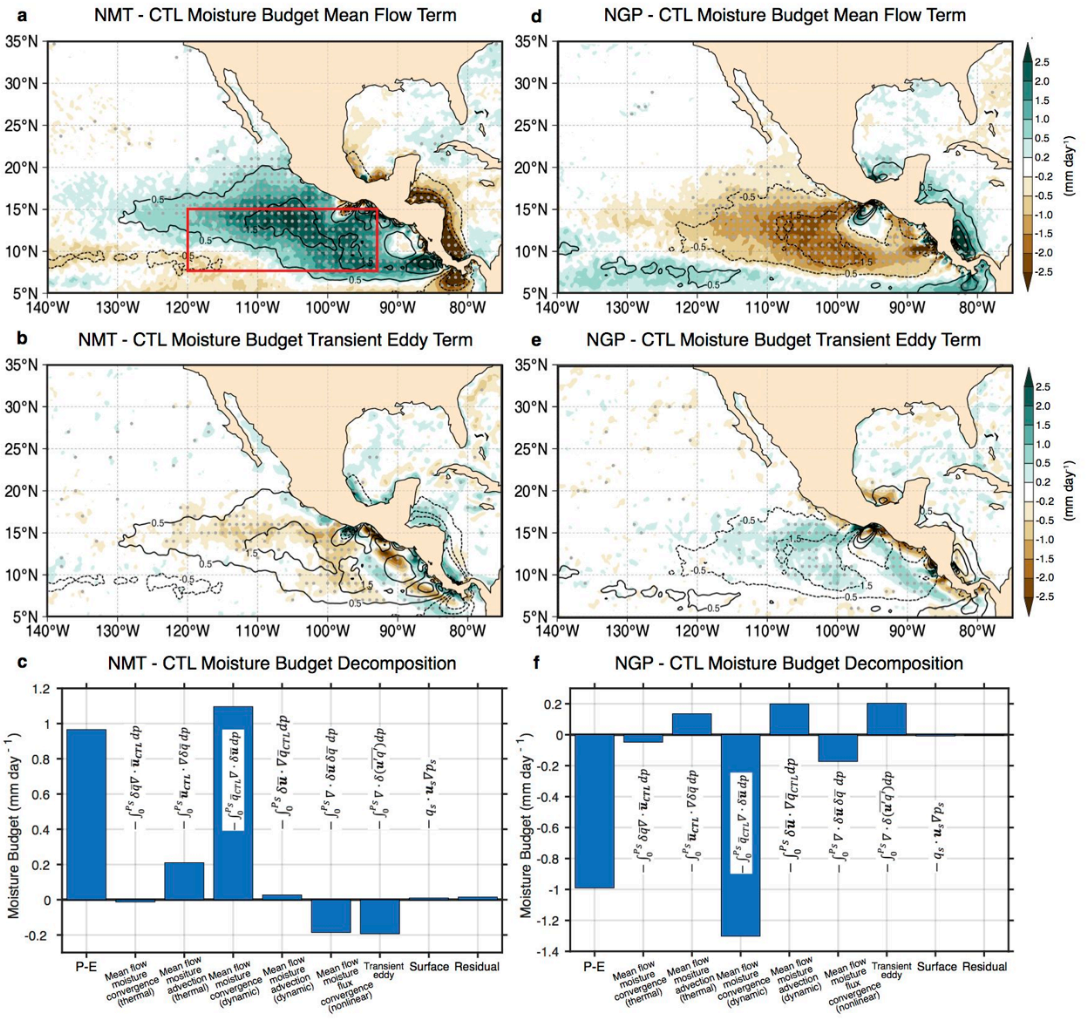

Supplementary Figure 6 | Moisture budget decompostion. The differences of mositure flux convergence a) by the mean flow ($-\frac{1}{g\rho_w}\int_0^{Ps}\nabla\cdot(\overline{\boldsymbol{u}}\overline{q})dp$; unit: mm day^{-1}; colorshadings) and b) by the transient eddy ($-\frac{1}{g\rho_w}\int_0^{Ps}\nabla\cdot(\overline{\boldsymbol{u}'q'})dp$; unit: mm day^{-1}; colorshadings) using the NMT minus CTL ensemble mean. The precipitation minus evaporation difference (P-E; mm day^{-1}) is overlaid in black contours (contour starting from -2.5 with the interval of 1; positive solid lines and negative dotted lines). c) Relative contributions of various moisture budget terms to changes in hydrological cycle averaged over the red box (120°W to 93°W, 8°N to 15°N) in panel a). Refer to Methods for details of moisture budget analysis and decomposition of moisture flux convergence. d-f) Similar to a-c), but for the NGP minus CTL. Grey dots denote statistical confidence at the 95% level based on the two-sided t-test.

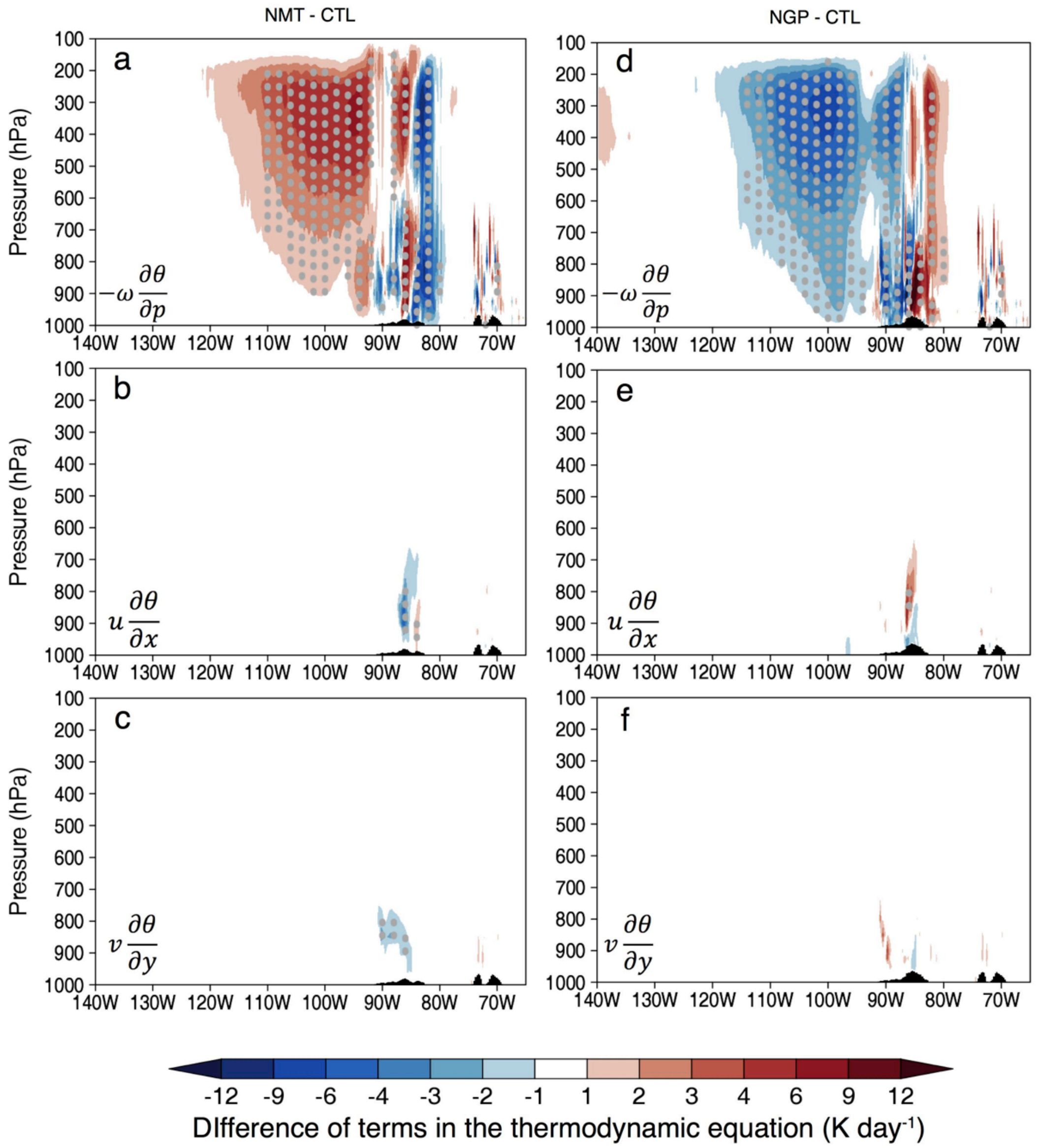

Supplementary Figure 7 | Simulated vertical profile of terms in thermodynamic energy equation over 8°N-15°N response to topography. Difference of a) vertical, b) zonal and c) meridional advection of potential temperature using the NMT simulation minus CTL simulation. d-f) Similar, but for the NGP simulation. Black shadings in a-c) and d-f) show the averaged surface pressure in the CTL and NGP. Note that the averaged surface pressure in NMT between 90°W and 80°W is above 1000 hPa, thus showing CTL for reference here. The difference in potential temperature tendency term is negligible and not shown here. Grey dots denote statistical confidence at the 95% level based on the two-sided t-test.

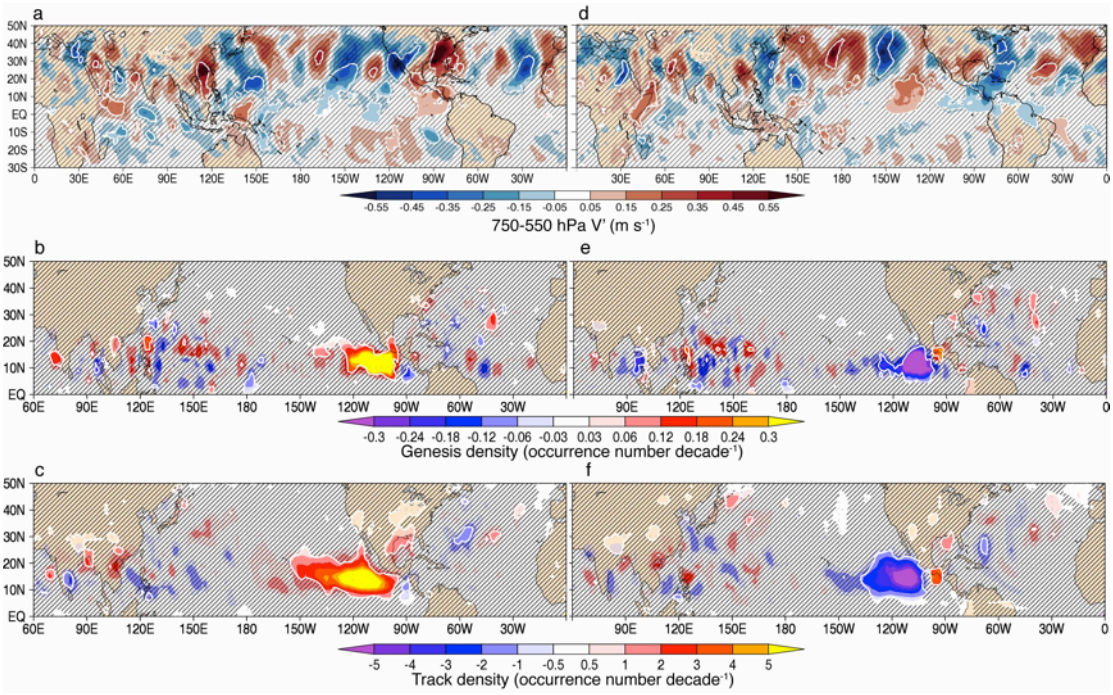

Supplementary Figure 8 | Intrabasin view of TC activity changes response to topography. The difference of a) zonally anomalous lower-tropospheric (750-550 hPa) meridional wind (m s^{-1}), b) TC genesis density (occurrence number $decade^{-1}$) and c) TC track density (occurrence number $decade^{-1}$) using the NMT minus CTL. d-f) Similar, but for the NGP simulation. Hatches denote the differences are not statistically confident at the 90% level based on the two-sided t-test.

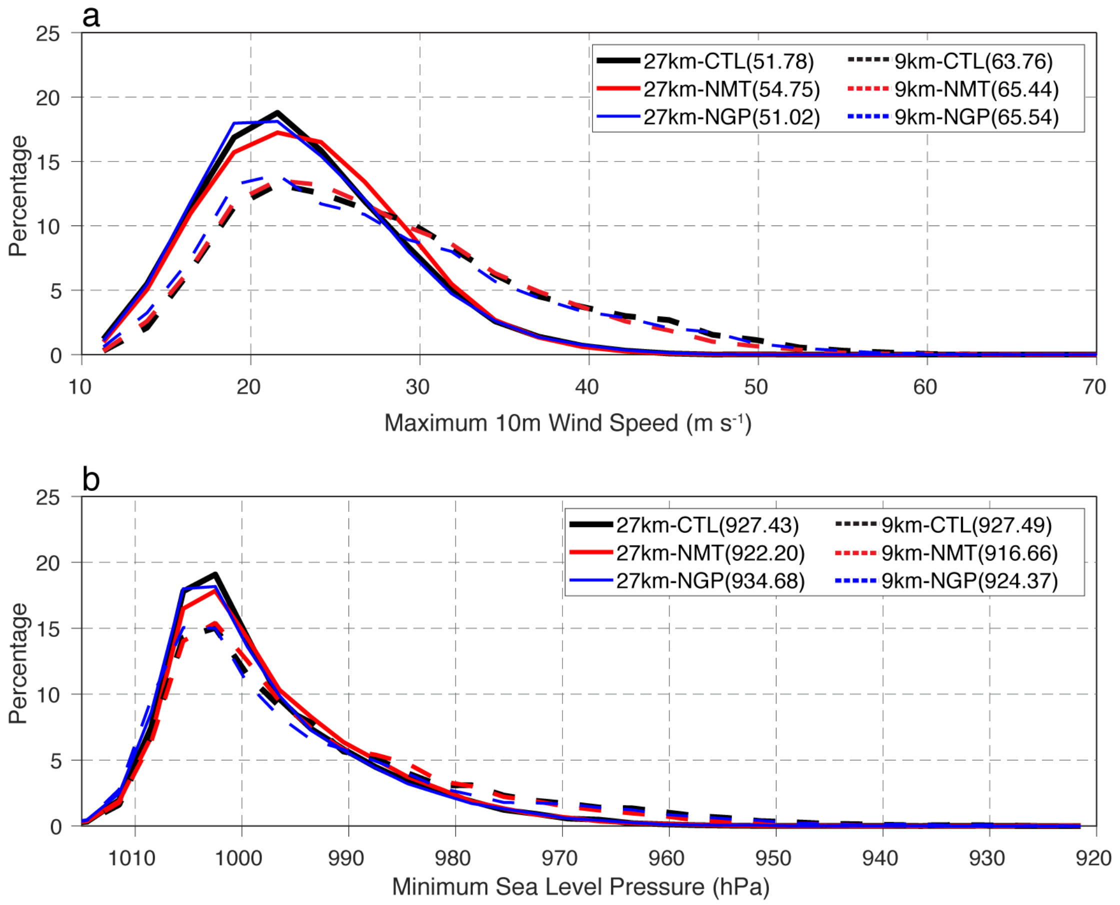

Supplementary Figure 9 | Probability distribution functions of the simulated ENP TC intensities. a) Maximum 10-m wind velocity (m s^{-1}) of TCs from the CTL (black solid line), NMT (red solid line), NGP (blue solid line), CTL9km (black dashed line), NMT9km (red dashed line), and NGP9km (blue dashed line). The peak intensity within each experiment is shown in the legend. b) Similar, but for TC intensity in terms of minimum sea level pressure (hPa).

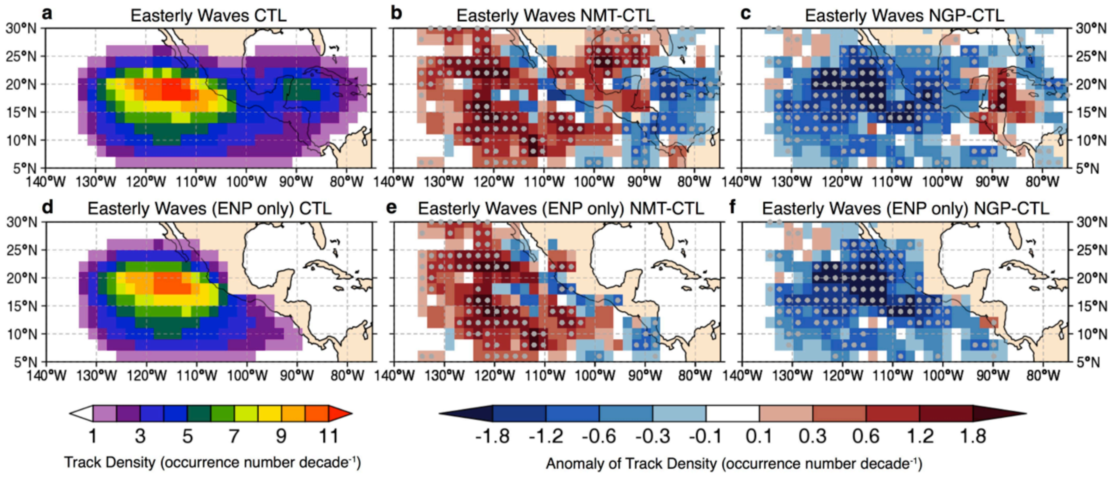

Supplementary Figure 10 | Easterly waves track density. a) The CTL ensemble mean of seasonal easterly wave track density (occurrence number decade^{-1}) and the differences of using g) NMT minus CTL and h) NGP minus CTL. d-f) Same as a-c), but for those easterly waves orignated in the ENP (not propagated from the Atlantic). Grey dots denote statistical confidence at the 95% level based on the two-sided t-test.
